# A regularization-free elasticity reconstruction method for ultrasound elastography with freehand scan

**DOI:** 10.1186/1475-925X-13-132

**Published:** 2014-09-07

**Authors:** Xiaochang Pan, Ke Liu, Jing Bai, Jianwen Luo

**Affiliations:** Department of Biomedical Engineering, School of Medicine, Tsinghua University, Beijing, 100084 China; Division of Electronics and Information Technology, National Institute of Metrology, Beijing, 100013 China; Center for Biomedical Imaging Research, Tsinghua University, Beijing, 100084 China

## Abstract

**Background:**

In ultrasound elastography, reconstruction of tissue elasticity (e.g., Young’s modulus) requires regularization and known information of forces and/or displacements on tissue boundaries. In practice, it is challenging to choose an appropriate regularization parameter; and the boundary conditions are difficult to obtain *in vivo*. The purpose of this study is to develop a more applicable algorithm that does not need any regularization or boundary force/displacement information.

**Methods:**

The proposed method adopts the bicubic B-spline as the tissue motion model to estimate the displacement fields. Then the estimated displacements are input to the finite element inversion scheme to reconstruct the Young’s modulus of each element. In the inversion, a modulus boundary condition is used instead of force/displacement boundary conditions. Simulation and experiments on tissue-mimicking phantoms are carried out to test the proposed method.

**Results:**

The simulation results demonstrate that Young’s modulus reconstruction of the proposed method has a relative error of −3.43 ± 0.43% and root-squared-mean error of 16.94 ± 0.25%. The phantom experimental results show that the target hardening artifacts in the strain images are significantly reduced in the Young’s modulus images. In both simulation and phantom studies, the size and position of inclusions can be accurately depicted in the modulus images.

**Conclusions:**

The proposed method can reconstruct tissue Young’s modulus distribution with a high accuracy. It can reduce the artifacts shown in the strain image and correctly delineate the locations and sizes of inclusions. Unlike most modulus reconstruction methods, it does not need any regularization during the inversion procedure. Furthermore, it does not need to measure the boundary conditions of displacement or force. Thus this method can be used with a freehand scan, which facilitates its usage in the clinic.

## Background

Breast cancer is the most common cancer among women, and the second-leading cause of cancer deaths in women in the United States [1]. The pathological state of the breast cancer highly correlates with their mechanical properties, such as Young’s modulus (or shear modulus) and viscoelasticity [2]. This lays the foundation of manual palpation routinely used in breast cancer detection. Palpation is especially helpful in the detection and localization of breast lesions [3]. However, it is limited in the cases when the tumor is small or deep beneath the skin surface [4]. Besides, palpation is subjective and only provides qualitative information. Based on the concept of palpation, quasi-static elastography (or compression elastography) is proposed to non-invasively estimate the mechanical property of soft tissues using ultrasound imaging [5].

In quasi-static elastography, the axial strain field is interpreted as relative Young’s modulus distribution within the tissue with the assumption of constant stress distribution [5]. The true Young’s modulus distribution could be computed from the internal strain and stress field [6]. However, the internal stress distribution cannot be measured *in vivo* [7]. Due to the non-uniform stress distribution within the tissue, several mechanical artifacts could exist in the axial strain image and may compromise the diagnosis in the clinic [8]. For examples, stress decay with depth causes the target-hardening artifact [5]. To overcome this limitation, many researchers are devoted to reconstructing the Young’s or shear modulus within the tissue by using certain constraints and the estimated displacements or strains [8–15].

The reconstruction of Young’s modulus is an inverse problem [8, 16]. Most inversion approaches assume that the tissue is linearly elastic, isotropic, continuous and incompressible [8, 11, 13–15]. In addition, the three-dimensional (3D) elasticity problem is usually simplified to a two-dimensional (2D) problem by assuming plane-strain [8] or plane-stress [14] conditions. The approaches for this inverse problem can be mainly divided into two categories, i.e., direct inversion approaches [14, 15, 17, 18] and iterative inversion approaches [8, 10, 16, 19–21].

The direct approaches compute the Young’s or shear modulus by solving the partial differential equations of equilibrium [7, 16]. These approaches rearrange the equilibrium equation used in the forward problem, and the Young’s modulus could be directly reconstructed by using special boundary conditions and estimated strain fields [16]. However, the boundary conditions are the known modulus values or pressures on the boundaries of the region of interest (ROI), which are difficult to measure *in vivo* [7]. In addition, the errors in the estimated strain field can be greatly amplified in the direct inversion, and thus may make the reconstruction result unreasonable [16].

The iterative approaches treat the inverse problem as a nonlinear optimization problem. The shear modulus distribution is calculated from minimization of the difference between the measured displacements and predicted displacements computed in the forward problem (i.e., the objective function) [7, 22]. Generally, the iterative inversion approaches are more robust than the direct inversion approaches [16]. However, these approaches also have some limitations. Firstly, they need to solve the forward problem several times, and hence are computationally expensive [16, 21]. Besides, if the objective function contains multiple minima, it may converge to a wrong solution rather than the true one [12]. Furthermore, the iterative inversion requires a regularization term. And choosing an appropriate regularization parameter is challenging [20]. The purpose of regularization is to suppress the reconstruction noise. The value of the regularization parameter can affect the size of inclusion shown in the reconstructed modulus image and the contrast between the inclusion and background [20]. An appropriate regularization parameter should be used to reduce the noise and preserve the contrast of the reconstructed modulus image simultaneously [20]. However, it is difficult to obtain the optimal regularization parameter in practice.

To the authors’ knowledge, current elasticity inversion schemes require complex equipment to obtain the boundary conditions of displacement or force [23–26]. These complex equipment limit the Young’s modulus reconstruction technique to be used in a freehand ultrasound scan for breast elastography. The purpose of this study is to develop an elasticity reconstruction method that does not need too much manual intervention or measurements of boundary conditions, which can be implemented in common medical ultrasound systems with a freehand scan. To overcome the limitations of the existing inversion approaches, a direct inversion scheme based on finite element method (FEM) is developed. It combines the advantages of direct and iterative inversion approaches. A novel feature of the proposed method is the utilization of the bicubic B-spline function as the tissue motion model to suppress the noise in the inversion, meanwhile it does not need any regularization in the inversion. Besides, a more practical modulus boundary condition is used in this method instead of force or displacement boundary condition. Simulations and phantom experiments are conducted to validate the effectiveness of the proposed method.

## Methods

### Inversion method

In the proposed inversion method of Young’s modulus reconstruction, the tissue was modelled as linearly elastic, isotropic, continuous and incompressible [8, 11, 13–15]. Besides, the commonly used plane-strain approximation is adopted in the proposed method. As the boundary forces of the reconstruction ROI are unknown, the reconstructed Young’s moduli are relative values (i.e., contrast) rather than absolute values for there is no known stress information [12]. The proposed method is comprised of two algorithms. The first algorithm is a bicubic B-spline fitting-based technique which estimates the 2D axial and lateral displacement fields from a pre-estimated axial displacement field, the plane-strain constraints and incompressibility assumption. The second algorithm is FEM-based direct inversion with modulus boundary condition. These algorithms are described in the following sections.

#### Displacement estimation

The displacements within the tissue are first estimated through a two-step optical flow method using ultrasound radiofrequency (RF) signals [27]. It has been proved that the displacements estimated from this method have a rather high accuracy. However, they cannot be directly used in the FEM-based inversion method, since the inversion results are very sensitive to the errors in the displacement measurements. The modulus reconstruction accuracy degrades rapidly when the standard deviation of the displacement error exceeds 10^−4^ mm [11]. However, the displacement errors are typically larger than this threshold [28]. Hence, the estimated displacements need to be processed before the inversion.

The B-spline fitting technique is used to smooth the displacements estimated from the optical flow method. We denote *a*_*i,j*_ with size of *n*_*x*_*×n*_*y*_ as axial displacement parameters of bicubic B-spline function, and *b*_*i,j*_ as lateral displacement parameters with the same size. *n*_*x*_ and *n*_*y*_ are the number of uniformly-spaced knots in the lateral and axial directions, respectively. The axial displacement field *V* and lateral displacement field *U* can be presented as
12

where *i* = [*x*/*n*_*x*_] − 1, *j* = [*y*/*n*_*y*_] − 1, *p* = *x*/*n*_*x*_ − [*x*/*n*_*x*_], *q* = *y*/*n*_*y*_ − [*y*/*n*_*y*_] and where *B*_*m*_ and *B*_*n*_ stands for the *m*-th and *n*-th basis function of the B-spline [29, 30], respectively.

The axial displacement parameters *a*_*i,j*_ are calculated by fitting the axial displacements *V*(x, y) of B-spline and estimated axial displacements *V*_*0*_ (*x*, *y*) from optical flow using the least square technique [31]. Hence, the axial displacements estimated with this method could preserve a rather high precision and smoothness. However, the lateral displacements estimated from optical flow are much noisier than axial displacements, due to lower resolution, lower sampling rate and lack of phase information in the lateral direction [32]. It is inappropriate to obtain the lateral displacement parameters *b*_*i,j*_ in a similar way as the axial parameters. To overcome this limitation, the constraints of 2D plane strain and the incompressibility of the tissue are utilized to compute the lateral displacement. These constraints could be formulated as.
3

where  and  denote the lateral and axial normal strains, respectively. As the axial strain  has been accurately estimated from the two-step optical flow method [27], and lateral strain  could be calculated with Eq. (3). Combining the known lateral strain  and the partial derivative of Eq. (2),
4

the lateral displacement parameters *b*_*i,j*_ can also be calculated by the least square technique [31]. Finally, the axial and lateral displacements of B-spline model could be computed by substituting *a*_*i,j*_ and *b*_*i,j*_ into Eqs. (1) and (2), respectively.

#### Elasticity reconstruction

**Figure 1:**
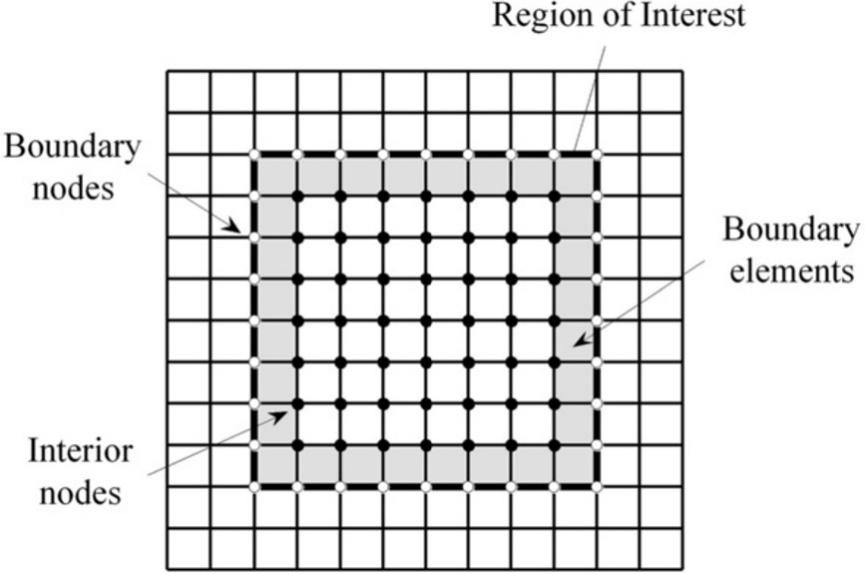
**An illustration of the 2D meshes with quadrilateral elements.** The ROI is outlined with thick lines. The boundary nodes are marked with white circles, and the interior nodes are depicted with black circles. The boundary elements are shown with the gray elements.

The estimated displacement field (including both axial and lateral components) is used as one of the inputs of the elasticity reconstruction algorithm. Nevertheless, the elasticity cannot be derived by the displacement field alone [33]. Either the stress distribution or the elastic modulus must be measured at a sufficient portion of the boundary [33]. As the internal geometry is unknown, the uniform quadrilateral elements are used in the FEM inversion model. The Poisson ratio is set to be 0.495 throughout the model under the near-incompressibility assumption [8]. The Young’s moduli of the boundary elements around the ROI are assumed to be the same in the model, and the Young’s moduli of the elements around the boundary of the ROI are set to unity (Figure 1).

The boundary node forces are first calculated by solving a 2D forward problem. By considering boundary elements as an object (Figure 1), the forces on the boundary nodes can be calculated by

5

where **K**_*boundary*_, **d**_*boundary*_ and **f** are the global stiffness matrix, the global nodal displacement vector and the global nodal force vector of the boundary, respectively. Node displacement **d**_*boundary*_ can be obtained from the estimated displacement field. The global stiffness matrix **K**_*boundary*_ is assembled from the elements with the known node position, Young’s modulus and Poisson ratio [34]. The global nodal force vector of boundary **f** determines the forces of the boundary nodes (white circles, Figure 1) and interior nodes (black circles, Figure 1).

After the boundary node forces have been calculated, the Young’s modulus distribution within the ROI is calculated by solving an inverse problem with FEM framework. Unlike the forward problem with known Young’s modulus distribution, the Young’s modulus of each element becomes unknown in the inverse problem. Hence, the Young’s modulus vector **E** is the variable to be solved. Now, consider all the elements within the ROI as an object. Denote the number of elements and nodes as *N*_*element*_ and *N*_*node*_, respectively. As described in [11], the Young’s modulus vector **E** can be extracted from the multiplication of the global stiffness matrix **K**_*roi*_ and the displacement vector **d**. The right term of Equation (5) becomes [11].
6

Here, **D** is a 2*N*_*node*_ × *N*_*element*_ matrix which is related to the elements and Poisson ratio, while the size of **E** is *N*_*element*_ × l. For more details about the procedure of assembly of matrix **D**, the readers are referred to [11]. For the quadrilateral element used in this method, *N*_*node*_ is greater than *N*_*element*_. The Young’s modulus vector **E** is solved by the least square method
7

The boundary nodal force vector **f**_*boundary*_ is a subset of **f** which has been solved in (5).

### Simulations

**Figure 2:**
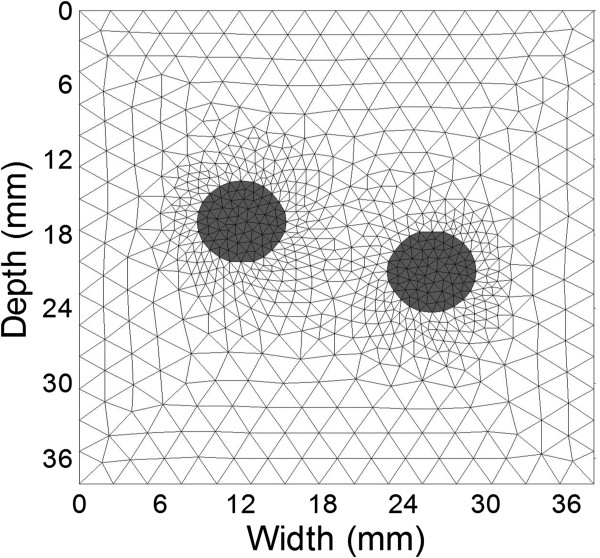
**The FE model used in the simulation study.** The two inclusions are shown with the gray meshes.

A 2D heterogeneous model with two circular inclusions embedded in a homogenous background was simulated in a plane strain condition (Figure 2). The model was assumed to be linearly elastic and nearly incompressible (with a Poisson ratio of 0.495) and had a size of 38 × 38 mm^2^ (axially × laterally, Figure 2). The inclusions had the same diameter of 6.5 mm. The Young’s moduli of the inclusions and background were 75 kPa and 25 kPa, respectively. Compressional axial strains between 0.5% and 2.0% were applied to the model, with a perfect slip boundary.

A 320-element linear array transducer with a center frequency of 6 MHz and a −3 dB bandwidth of 50% was simulated with Field_II software (http://field-ii.dk/) [35, 36]. The speed of sound was assumed to be 1540 m/s, and the sampling frequency of the RF signals was 32 MHz. The pitch of the transducer was 0.12 mm. The focal depth was 19 mm and the F number was 0.5. These transducer parameters were chosen to correspond to the actual transducer used in the phantom experiments. 320 lines were simulated in each ultrasound image, and the distance between adjacent lines was equal to the pitch. The imaging zone had a depth of 38 mm and a width of 38 mm. Randomly distributed scatterers were simulated within the model. The scatterer density was 200/mm^2^, which fulfilled the requirement for fully developed speckle in the simulated ultrasound image [37]. The pre-deformed RF signals of the model were simulated from Field_II using the parameters described above. Finite element analysis was performed with FEMLAB 2.3 software (Comsol Inc. Burlington, MA, USA) and MATLAB 6.5 (The MathWorks Inc., Natick, MA, USA) to calculate the theoretical displacements of the model. The scatterers were moved according to finite element solutions with different applied strains. The post-deformed RF signals were then simulated from Field_II using the new scatterers’ distribution. Gaussian white noises were added in the simulated RF signals, with a signal-to-noise ratio (SNR) of 30 dB.

### Phantom experiments

Experiments were performed on a tissue-mimicking elasticity QA phantom (Model 049A, CIRS Inc., Norfolk, VA, USA) and a breast elastography phantom (Model 059, CIRS Inc.). For the elasticity QA phantom, the Young’s moduli of the background and cylindrical inclusions were 25 ± 6 kPa and 80 ± 12 kPa (mean ± standard deviation), respectively. There were multiple cylindrical inclusions in the phantom and those used in the experiments had diameters between 2.5 and 10.4 mm. For the breast elastography phantom, 5 spherical inclusions of different sizes were randomly distributed within the phantom. And the Young’s moduli of the inclusions were at least two times greater than that of the background.

**Figure 3:**
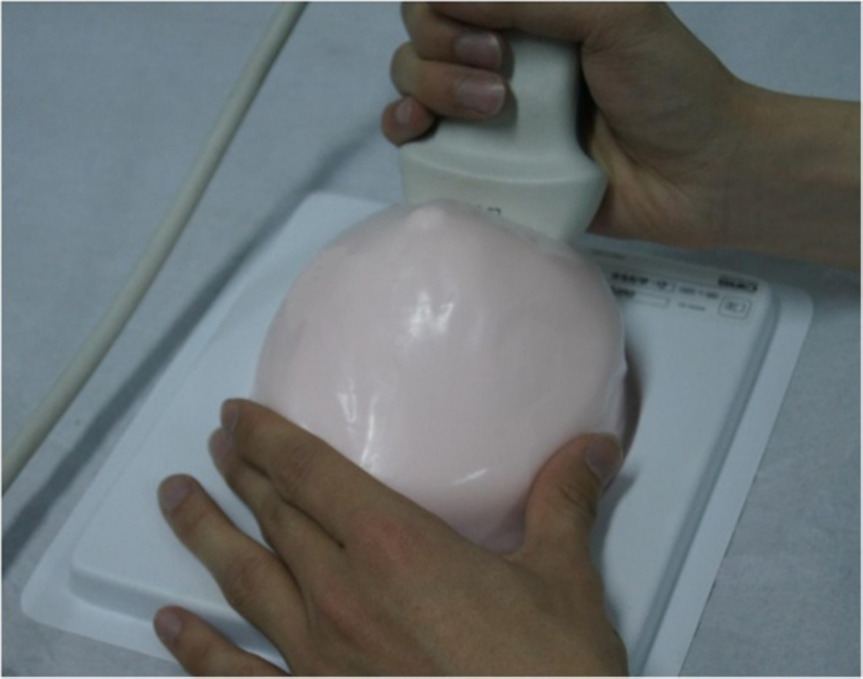
**The tissue-mimicking breast phantom used in the phantom study.** The acquisition of ultrasound RF data of the breast phantom with freehand scan.

The ultrasound RF signals of the phantoms were recorded at a sampling frequency of 32 MHz and a frame rate of 91 Hz using a Philips iU22 ultrasound system (Philips Medical Systems, Bothell, WA, USA), equipped with an L9-3 linear array transducer. Each image consists of 320 lines, with a distance of 0.12 mm between adjacent lines. The RF data were acquired from the pre- and post-deformed phantom with freehand scan, as shown in Figure 3.

### Quantitative evaluation

In the optical flow-based displacement estimation, a window size of 2.2 × 1.8 mm^2^ (height × width) was used. In the following B-spline fitting, the axial and lateral knot spacing were 4.82 mm and 4.75 mm, respectively. For the modulus reconstruction, the quadrilateral element used in the inversion was 0.48 × 0.48 mm^2^ (height × width). These parameters were used in the data processing of both simulation and phantom studies. The displacement estimation and elasticity reconstruction methods described above were used in both the simulation and phantom studies.

In the simulation study, as the ground true is available, the mean relative error [11] and root-mean-square error (RMSE) of the relative Young’s modulus were utilized to evaluate the accuracy of the Young’s modulus reconstruction. Here, the mean relative error and RMSE are defined as follows
89

## Results

### Simulation results

**Figure 4:**
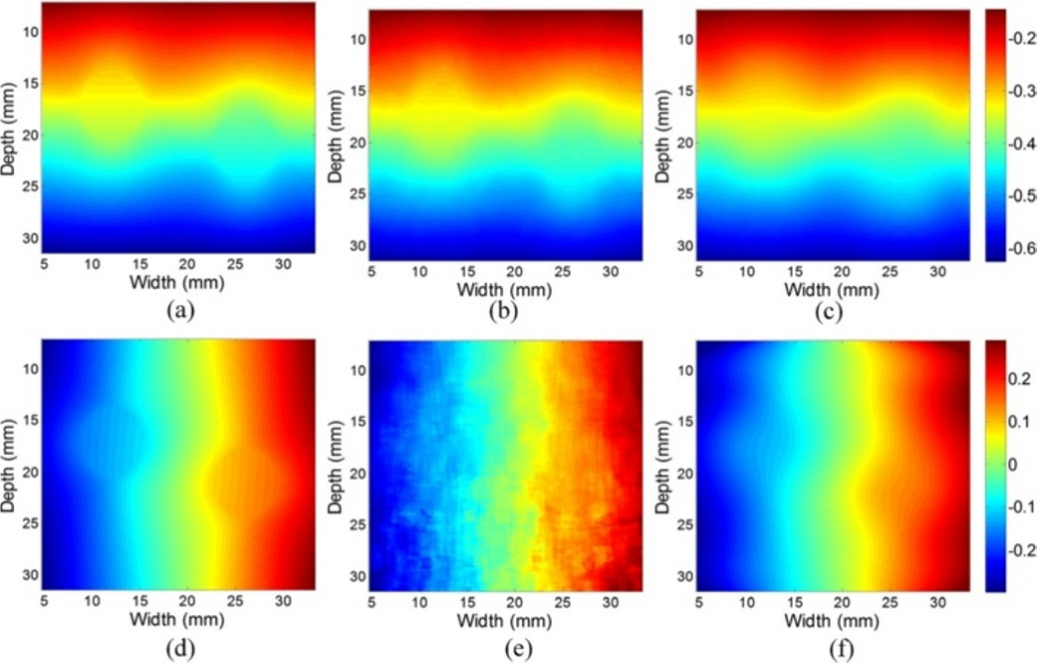
**The estimated displacements at an applied strain of 2.0%** **.** The theoretical **(a)** axial and **(d)** lateral displacement. The **(b)** axial and **(e)** lateral displacement estimated from optical flow. The **(c)** axial and **(f)** lateral displacement computed from B-spline fitting.

Figure 4 compares the displacements estimated from the optical flow method (Figures 4(b) and (e)) and from B-spline fitting (Figures 4(c) and (f)) with the theoretical displacements (Figures 4(a) and (d)). The axial displacements estimated from optical flow (Figure 4(b)) are closer to the theoretical axial displacements than B-spline fitting (Figure 4(c)). The standard deviations of axial displacement errors of optical flow and B-spline fitting are 1.9 × 10^−3^ and 2.6 × 10^−3^ mm, respectively. The lateral displacements obtained from B-spline fitting (Figure 4(f)) are smoother than those estimated from optical flow (Figure 4(e)), and closer to the theoretical displacement (Figure 4(d)). The standard deviations of lateral displacement errors of optical flow and B-spline fitting are 13.4 × 10^−3^ and 6.9 × 10^−3^ mm, respectively. This suggests that the lateral displacements estimated from B-spline fitting are more precise than those from optical flow.

**Figure 5:**
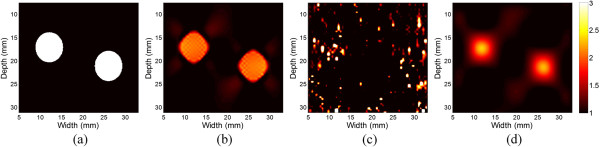
**The estimated Young’s modulus distributions. (a)** The theoretical Young’s modulus distribution. **(b)** The Young’s modulus reconstructed from the theoretical displacements calculated from finite element analysis. **(c)** The Young’s modulus reconstructed from the estimated displacements from optical flow. **(d)** The Young’s modulus reconstructed from the estimated displacements of B-spline fitting.

Figure 5 shows the theoretical Young’s modulus distribution (Figure 5(a)) and the Young’s modulus distributions reconstructed from theoretical displacements (Figure 5(b)) and from displacements estimated from optical flow (Figure 5(c)) and B-spline fitting (Figure 5(d)). Only relative Young’s moduli (i.e., contrast) are shown because the stress is unknown (the same below). The quadrilateral mesh used in the reconstruction has a size of 0.48 × 0.48 mm^2^ (height × width). Due to the noise in the lateral displacements estimated from optical flow (Figure 4(e)), the modulus could not be correctly reconstructed (Figure 5(c)). The Young’s moduli of the inclusion reconstructed from both the theoretical and estimated displacements are lower than the theoretical value. The Young’s moduli reconstructed from the theoretical displacement were relatively uniform within the inclusions, with a rapid transition from the inclusion to the background (Figure 5(b)). In contrast, the Young’s moduli reconstructed from the estimated displacement have the highest values in the center of the inclusions and gradually decrease with the distance from the center. A smooth transition from the inclusion to the background is shown (Figure 5(d)).

**Figure 6:**
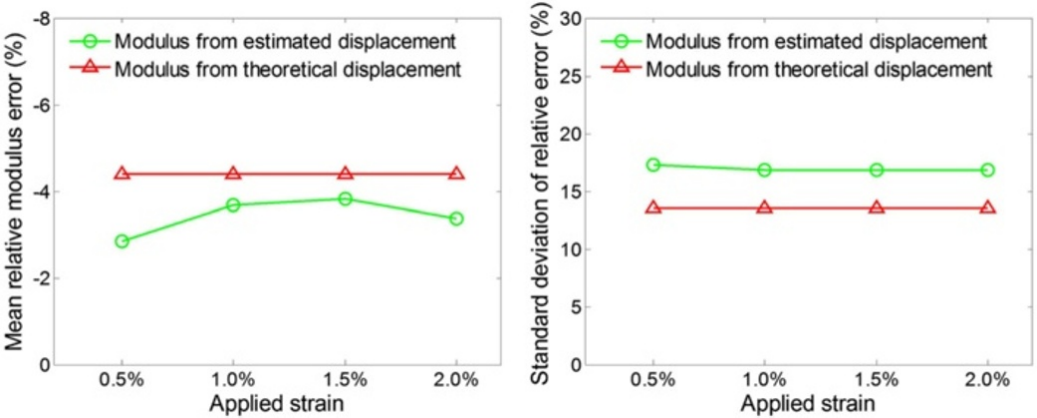
**Modulus reconstruction error analysis.** Comparison of the errors of reconstructed Young’s moduli from the theoretical and estimated displacements.

To quantitatively investigate the error of Young’s modulus reconstruction, the mean relative error and RMSE between the theoretical and reconstructed modulus values are calculated with different applied strains (Figure 6). The mean relative errors of reconstructed Young’s modulus from the estimated displacements of B-spline model is −3.43 ± 0.43% and is about 22% smaller than those from the theoretical displacements (−4.40%). The RMSE of the modulus estimates of B-spline displacements is 16.94 ± 0.25% and is about 25% larger than those of theoretical displacements (13.52%). These can be explained by the small errors in the estimated displacements.

### Phantom results

**Figure 7:**
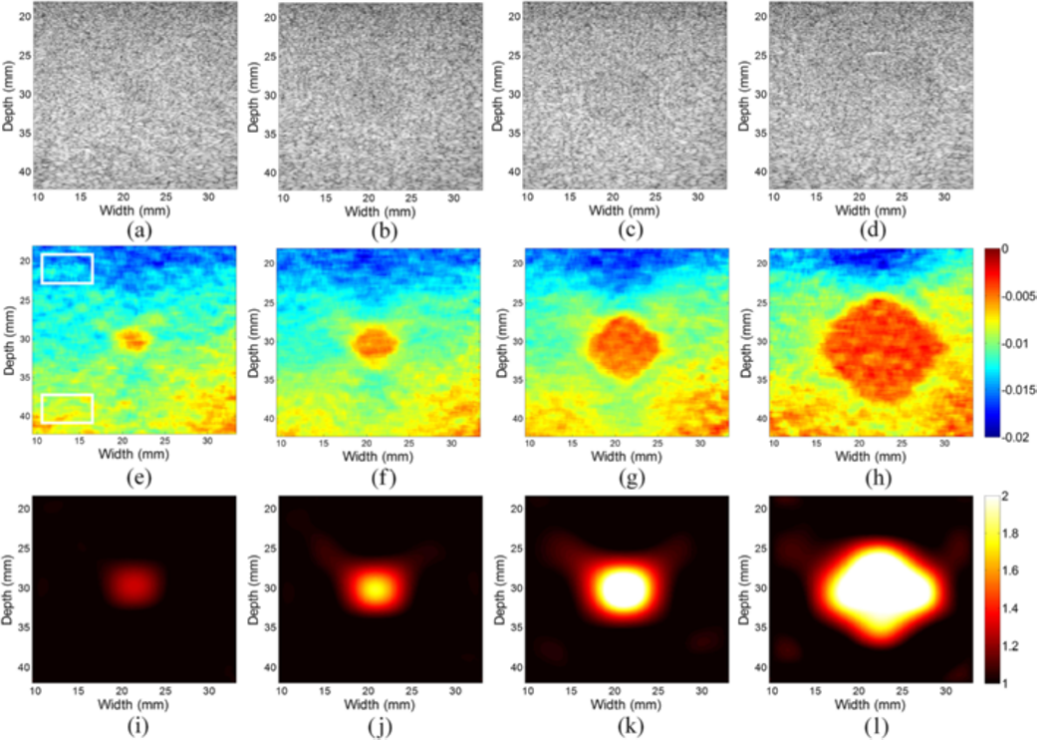
**Modulus reconstruction results of inclusions with different diameters. (a)**-**(d)** The B-mode images of inclusions with diameters ranging from 2.5 mm to 10.4 mm. **(e)**-**(h)** The corresponding axial strain images at an applied strain of 1.0%. **(i)**-**(l)** The corresponding reconstructed Young’s modulus images.

Figure 7 demonstrates the axial strain images and Young’s modulus images of different inclusions in the tissue mimicking elasticity QA phantom. The diameters of inclusions are 2.5 mm, 4.1 mm, 6.5 mm and 10.4 mm, respectively, and inclusions are located in the middle of the ultrasound image plane, as can be roughly found in the B-mode images (Figures 7(a)–(d)). The inclusion’ locations in the axial strain images (Figures 7(e)–(h)) and Young’s modulus images (Figure 7(i)–(l)) are in a good agreement with the B-mode images. However, the target hardening artifacts appear in the axial strain images, while the Young’s modulus images are free of these artifacts. And the reconstructed modulus image has a high spatial resolution of 2.5 mm (Figure 7(i)).

To quantitative investigate the target hardening artifacts, two rectangular ROIs from the top and bottom of axial strain images are chosen (Figure 7(e)), respectively. The estimated strains in the ROIs on the top and bottom are −1.39 ± 0.1% and −0.85 ± 0.08%, respectively. The strains in the ROI on the bottom are 39% lower than those on the top. In contrast, the estimated Young’s modulus values in the ROIs on the top and bottom are 1.00 ± 0.01 and 0.94 ± 0.01, respectively. The estimated Young’s modulus values in the ROI on the bottom are only 6% lower than those on the top. These suggest that the Young’s modulus images significant reduce the target hardening artifacts compared to the axial strain images.

**Figure 8:**
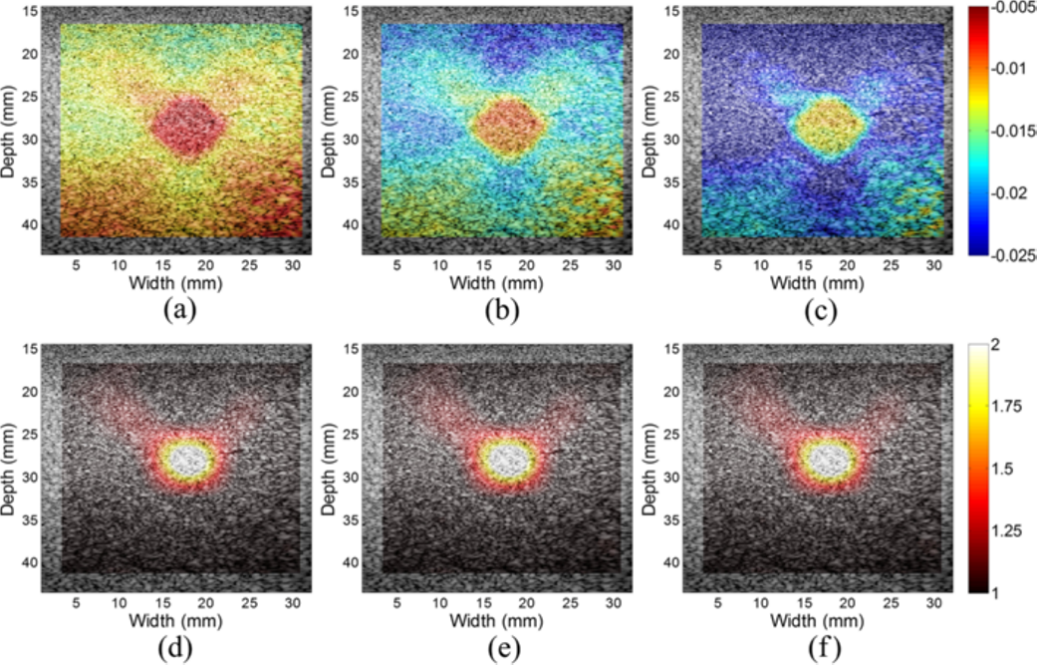
**The reconstructed Young’s modulus values to the different amount of applied forces. (a)**-**(c)** The estimated axial strain images overlaid onto the B-mode images under the applied strains of 1.14%, 1.70% and 2.26%, respectively. **(d)**-**(f)** The corresponding reconstructed Young’s modulus images overlaid onto the B-mode images.

To investigate the sensitivity of the modulus reconstruction results to the amount of applied force. The inclusion with a diameter of 6.5 mm in the elasticity QA phantom was chosen for this study. Different strains between from 1.14% and 2.26% were applied to the phantom. The applied strains were calculated by the difference between the displacements on the top and bottom of the ROI. Figure 8 depicts the axial strain and Young’s modulus images overlaid on the B-mode image. The axial strains of the inclusion and background vary with the applied strains, while the reconstructed Young’s moduli are stable at different strains. The axial strain is related to both the tissue mechanical property and the external force magnitude, and the change of the external force will change the values of the axial strain correspondingly (Figure 8 (a)-(c)). In contrast, the Young’s modulus image reflects the intrinsic mechanical property of the tissue and is not affected by the external force (Figure 8 (d)-(f)). These results show that the propose method is not sensitive to the amount of applied forces.

**Figure 9:**
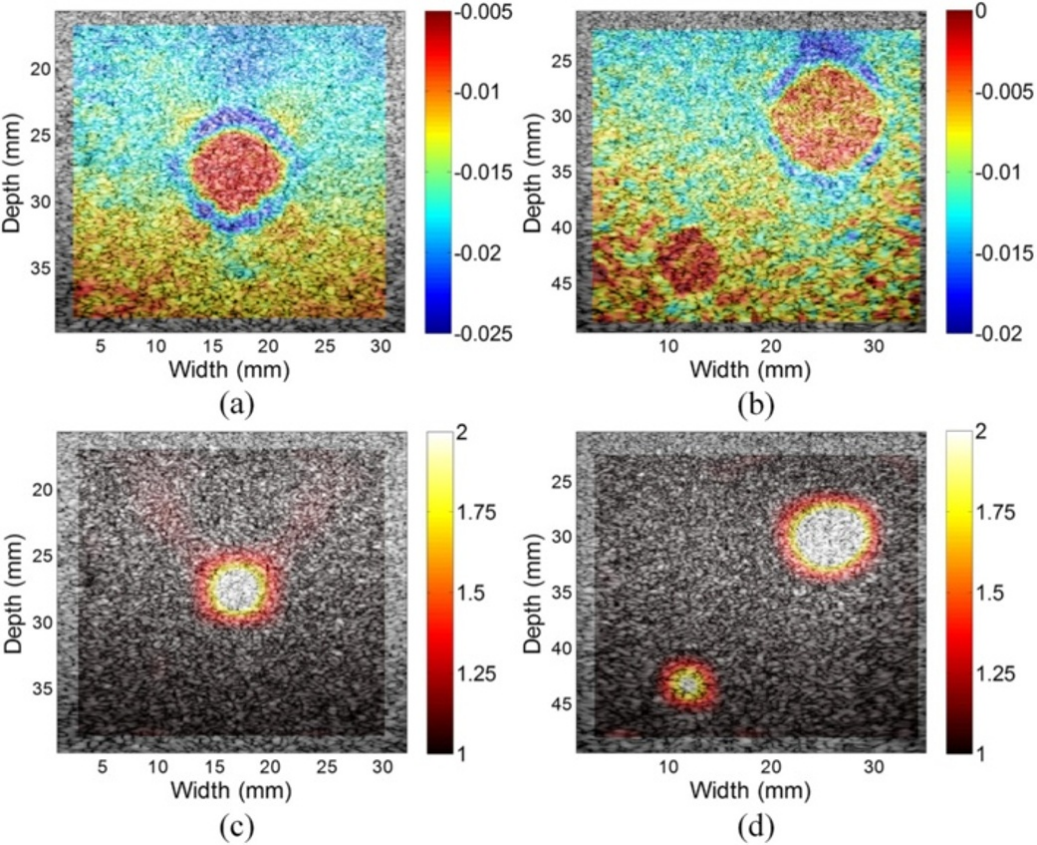
**The reconstructed Young’s modulus distributions of the breast phantom. (a)** The axial strain image and **(c)** Young’s modulus image of one inclusion overlaid onto the corresponding B-mode images. **(b)** The axial strain image and **(d)** Young’s modulus image of two inclusions overlaid onto the corresponding B-mode images.

For the breast elastography phantom, ultrasound RF data were acquired with freehand scan. Figure 9 shows the axial strain and reconstructed Young’s modulus images with one and two inclusions, respectively. In the Young’s modulus images, the locations and sizes of the inclusions match well to those in the axial strain images. Besides, the target hardening artifacts shown in the axial strain images are eliminated in the Young’s modulus images.

**Figure 10:**
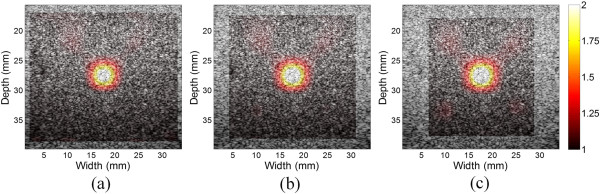
**The reconstruction Young’s modulus distributions of the breast phantom with different ROI sizes.** The different ROI sizes are **(a)** 24.1 × 33.2 mm^2^, **(b)** 23.1 × 28.5 mm^2^ and **(c)** 22.2 × 23.7 mm^2^.

Figure 10 shows the Young’s modulus images of the breast phantom with one inclusion using different ROI sizes in the reconstruction. The ROIs have sizes of 24.1 × 33.2 mm^2^ (height × width, Figure 10(a)), 23.1 × 28.5 mm^2^ (Figure 10(b)) and 22.2 × 23.7 mm^2^ (Figure 10(c)), respectively. The numbers of elements used in are 50 × 70, 48 × 60 and 46 × 50, respectively, and the computational time are 2.2 s, 1.2 s and 0.75 s, respectively. All the programs were performed on the platform of MATLAB 6.5 (The MathWorks Inc., Natick, MA, USA). The reconstructed modulus values of a circular ROI in the inclusion center with a radius of 2 mm are 1.87 ± 0.16, 1.98 ± 0.17 and 2.02 ± 0.18 for Figures 10(a), 10(b) and 10(c), respectively. Although the reconstruction ROIs are different in size, the estimated Young’s modulus distributions of the inclusion are similar.

## Discussion

The goal of this study is to develop a robust elasticity reconstruction method for ultrasound elastography with freehand scan. Conventional elastography typically shows axial strain images [3, 38], which suffer from mechanical artifacts (such as target hardening and stress concentration) and lack of quantitative information [7, 8]. The reconstruction of Young’s modulus or shear modulus (i.e., solving the inverse problem) can overcome these drawbacks. However, the inverse problem generally has an ill-posed nature [9, 25, 33]. In this work, we proposed a B-spline function-based displacement estimation method, and employed a modulus boundary condition to convert the ill-posed problem to a well-posed one. Simulations and phantom experiments were conducted to validate the performance of the proposed method. In the simulation study, the mean relative error and RMSE were used to evaluate the accuracy and precision of Young’s modulus reconstruction. In the phantom study, the reconstructed modulus images were compared with the corresponding strain images.

The axial and lateral displacement fields used in the inversion scheme are defined as bicubic B-spline function. This tissue motion model is also called free-form deformation [39], and has been used in ultrasound cardiac motion estimation [40, 41]. The conventional motion estimation methods are usually based on local information (Figures 4(b) and (e)), while the proposed B-spline fitting method takes the global information into consideration, and can obtain much smoother displacement estimation (Figures 4(c) and (f)). Although the axial displacement estimated from the optical method is more precise than that of the B-spline fitting method, they cannot be directly used in the inversion since the noise in the displacement will be amplified without the restriction of continuity. The B-spline displacement model has the implicit continuous constraint, and it is helpful to reduce the ill-condition of the Young’s modulus inversion. The tissue incompressibility and plane strain assumptions are used to estimate the lateral displacement, which is a necessary part for modulus reconstruction. The B-spline motion model could be implemented by the proposed displacement fitting or non-rigid registration of the RF signals [42]. However, the non-rigid registration is much more computationally expensive than displacement fitting, and may present local minima during the iteration process, due to the highly oscillatory nature of the RF signals [20]. Thus, the displacement fitting technique is used in this study.

Conventional ultrasound elastography typically shows strain images, and can provide improved ability to determine the lesions’ location and shape when compared to the corresponding B-mode image [3]. Nevertheless, under the assumption of stress uniformity, the strain image suffers from several artifacts and therefore do not reveal the true tissue elastic properties [8, 9]. In reality, the stress together with strain attenuates with depth, hence the tissue on the bottom part of the tissue seems stiffer than that on the top (Figures 7(c)-(h)). These are the so-called target hardening artifacts [5, 8, 9]. In the Young’s modulus images, these artifacts are eliminated (Figures 7(i)-(l), and Figures 9(c)-(d)), which may facilitate the diagnosis of the breast tumors in the clinic. It would have a high contrast of modulus image for the inclusion by fixing the scale of the image colour bar around 2, since the modulus contrast between normal and cancerous tissues is usually great than 100% [43].

The boundary conditions currently used in the iterative inversion schemes include displacement boundary conditions [13, 25], force boundary conditions [8] and a combination of both [16, 20, 23]. However, the force distribution within the tissue is difficult to measure *in vivo* [7]. The combination boundary conditions of displacement and force need special experimental setup [20, 23], and hence limit the usage in the clinic. The boundary condition used in the proposed method is the modulus boundary condition which assumes the moduli of the boundary elements around the reconstruction ROI are the same [13, 15, 44]. It may induce some artifacts if the moduli on the ROI boundary are different. However, the ROI around the inclusion could be chosen according to the B-mode and/or strain images in order to avoid this situation. Besides, the size or shape of the ROI can be changed. If the moduli of the inclusion do not have much difference when different ROIs are used, as shown in Figure 10, the modulus reconstruction result can be deemed reliable.

In the simulation study, the RMSE of the Young’s moduli reconstructed from the estimated displacements are about 25% higher than those from theoretical displacements, while the mean error of estimated Young’s moduli reconstructed from the estimated displacements are about 22% lower than those from the theoretical displacements (Figure 6). These results suggest that the precision of the Young’s modulus reconstructed by the proposed method is comparable to those from the theoretical displacements. As shown, the modulus distributions reconstructed from the theoretical displacements are not the same as the theoretical modulus distribution (Figures 5(a) and (c)). This can be explained by the measurement noise (i.e., displacement estimation noise) and process noise caused by the mismatch between the models used in the forward and inverse problems, which are the two main factors that affect the modulus reconstruction results [45]. In the forward problem, the object was meshed with non-uniform size elements, and the meshes were finer near the inclusion boundary (Figure 2). In the inverse problem, as the internal geometry of the tissue is usually unknown, the uniform quadrilateral elements were used (Figure 1). The discrepancy of the meshes used in the forward and inverse problems leads to reconstruction error even if the theoretical displacements are used as the input. The reconstruction errors in the simulation study are similar to the results of Zhu et al. [11].

Another limitation of the proposed method is the smoothing effect on the interface between the inclusion and background. This is mainly attribute to the smoothness of displacements by the bicubic B-spline fitting in the displacement estimation. The high-frequency estimation noise together with high-frequency displacement information is filtered out, and hence the high-frequency components in the modulus distribution are also eliminated. The smoothness of displacements used in the proposed method is similar to the regularization used in the iterative inversion schemes, which also tends to smooth over the sharp dips in the reconstructed modulus image [2, 16, 24, 25]. The lower reconstructed Young’s modulus values in the inclusions (or contrasts) are consistent with the results of a previous study [24]. As shown in Figure 7, the smaller the size of the inclusion is, the lager the influence of the smoothing effect will be. Hence, the result of the reconstructed Young’s modulus becomes worse when the inclusion gets smaller (Figure 7). To overcome these limitations, the mesh adaptation algorithm has been proposed to improve the accuracy of Young’s modulus reconstruction [46]. Nevertheless, the adaptive mesh generation requires image segmentation using the internal tissue geometry or axial strain information.

Tissue deformation is typically 3D in nature, but ultrasound RF data are often collected from a 2D imaging plane. The 2D plane strain simplification is utilized in the proposed method. Some errors may be incurred when the 3D elasticity problem is modelled by either plane strain or plane stress approximation [7, 20]. However, we believe that plane strain is a reasonable assumption for breast elastography in the clinic because it has proved that the motion perpendicular to the image plane is small in the breast *in vivo* [11]. And when 3D data are available, the FEM-based inversion scheme could be extended to 3D case. As a final note, the displacement fields could also be estimated from other imaging modalities such as magnetic resonance imaging (MRI), and the Young’s modulus distribution of tissue could be inferred by using the proposed method.

## Conclusions

In this paper, a finite element based Young’s modulus reconstruction method is proposed to facilitate the quantitative investigation of the elasticity of soft tissues. The B-spline function fitting method is proposed to reduce the displacement estimation noise. Then the Young’s modulus distribution within the tissue is reconstructed based on the framework of FEM with the input of displacements and modulus boundary condition. The simulation and phantom studies demonstrated that the proposed method yields the inclusion’s relative modulus with reasonably high accuracy, and correctly delineates the locations and sizes of inclusions. Besides, the computational time of the proposed reconstruction method ranges from 0.75 to 2.2 s, which is much faster than the conventional iterative reconstruction methods (e.g., 18 hours) [21]. More importantly, the proposed method does not need any regularization or boundary force information, thus avoiding too much manual intervention and special equipment. With these advantages, the proposed method could be implemented in conventional ultrasound systems with freehand scan.
